# Supplementary material for: Screening coffee genotypes for brown eye spot resistance in Brazil
Source: PLoS One. 2022 Jan 31;17(1):e0258822. doi: 10.1371/journal.pone.0258822 (PMC8803159; doi:10.1371/journal.pone.0258822)
Supplement: S1 Table — PR: partially resistant, MS: moderately susceptible, S: susceptible, AS: highly susceptible. *Cultivar used as a control. CV–Catura Vermelho; HT–Híbrido de Timor h2m: heritability of genotype averages. h2i: broad-sense heritability individually for each plot, or the total genotypic effects. c2perm: coefficient of determination of the permanent environmental effects. MO: Genotypic mean of 60 genotypes before selection. MS Mean of the five selected genotypes. SG: gain selection. (DOCX) [file pone.0258822.s001.docx]

**S1 Table: Genotypes and their respective distribution of leaf lesions frequency into six classes, class 1: 0.1-3.0; class 2: 3.1-6.0; class 3: 6.1-12.0; class 4: 12.1-18.0; class 5: 18.1-30.0; class 6: 30.1-50% of the leaf surface affected by brown eye spot, phenotypic mean of the genotypes (M), classification (C) of the accessions based on the phenotypic mean, predicted additive breeding values (u + g), heritability (H) of genotypes, genetic parameters, general mean and estimated gain relative to the severity of brown eye spot in 5 selected genotypes.** PR: partially resistant, MS: moderately susceptible, S: susceptible, AS: highly susceptible. *Cultivar used as a control. CV – Catura Vermelho; HT – Híbrido de Timor h^2^_m_: heritability of genotype averages. h^2^_i_: broad-sense heritability individually for each plot, or the total genotypic effects. c^2^_perm_: coefficient of determination of the permanent environmental effects. M_O_: Genotypic mean of 60 genotypes before selection. M*_S_* Mean of the five selected genotypes. SG: gain selection.

| **Access** | **Genotype** | | | **1** | **2** | **3** | | **4** | **5** | | **6** | **M** | **C** | | **u + g** | **H** |  |
| --- | --- | --- | --- | --- | --- | --- | --- | --- | --- | --- | --- | --- | --- | --- | --- | --- | --- |
| MG 1207 | Sumatra | | | 0 | 100 | 0 | | 0 | 0 | | 0 | 4.6 | PR | | 2.37 | 0.88 |  |
| - | Paraíso MG H 419-1 | | | 0 | 0 | 100 | | 0 | 0 | | 0 | 6.8 | MS | | 3.21 | 0.82 |  |
| - | Asabranca | | | 0 | 0 | 0 | | 100 | 0 | | 0 | 12.1 | S | | 5.23 | 0.81 |  |
| MG 0321 | HT UFV 432-09 | | | 0 | 0 | 0 | | 100 | 0 | | 0 | 12.8 | S | | 5.37 | 0.78 |  |
| MG 0132 | Sumatra Amarelo Pl 01 | | | 0 | 0 | 0 | | 100 | 0 | | 0 | 12.1 | S | | 5.49 | 0.77 |  |
| MG 0176 | Amphillo x H. Natural MR 36-349 | | | 0 | 0 | 0 | | 100 | 0 | | 0 | 13.6 | S | | 5.85 | 0.78 |  |
| - | Sacramento MG-1 | | | 0 | 0 | 0 | | 100 | 0 | | 0 | 13.6 | S | | 6.04 | 0.78 |  |
| MG 0280 | Híbrido Timor UFV 376-14 | | | 0 | 0 | 0 | | 100 | 0 | | 0 | 15.1 | S | | 6.52 | 0.79 |  |
| MG 0380 | HT UFV 445-70 | | | 0 | 0 | 0 | | 0 | 75 | | 25 | 31.5 | AS | | 10.28 | 0.86 |  |
| MG 0265 | Durandé Arabica x Canephora | | | 0 | 0 | 0 | | 0 | 75 | | 25 | 31.5 | AS | | 12.89 | 0.72 |  |
| MG 0279 | HT UFV 376-31 | | | 0 | 0 | 0 | | 0 | 75 | | 25 | 31.5 | AS | | 13.52 | 0.78 |  |
| MG 0324 | HT UFV 433-01 | | | 0 | 0 | 0 | | 0 | 75 | | 25 | 31.5 | AS | | 14.46 | 0.72 |  |
| MG 0267 | HT UFV 377-34 | | | 0 | 0 | 0 | | 0 | 75 | | 25 | 31.5 | AS | | 15.76 | 0.75 |  |
| MG 0723 | CV x CIFC H79/1UFV 339-02 | | | 0 | 0 | 0 | | 0 | 100 | | 0 | 30 | AS | | 15.9 | 0.79 |  |
| MG 0333 | HT UFV 437-10 | | | 0 | 0 | 0 | | 0 | 25 | | 75 | 34.5 | AS | | 16.16 | 0.74 |  |
| MG 0134 | Sumatra Palma | | | 0 | 0 | 0 | | 0 | 75 | | 25 | 31.5 | AS | | 17.15 | 0.73 |  |
| **-** | Araponga MG1 | | | 0 | 0 | 0 | | 0 | 100 | | 0 | 30 | AS | | 17.68 | 0.8 |  |
| MG 0179 | Catuaí Vermelho x Amphillo MR 2161 | | | 0 | 0 | 0 | | 0 | 0 | | 100 | 36.1 | AS | | 17.75 | 0.74 |  |
| - | Catucaí Amarelo 2SL | | | 0 | 0 | 0 | | 0 | 0 | | 100 | 36.1 | AS | | 17.88 | 0.88 |  |
| MG 0282 | HT UFV 376-12 | | | 0 | 0 | 0 | | 0 | 100 | | 0 | 30 | AS | | 19.42 | 0.88 |  |
| MG 0663 | CV x CIFC H276/2UFV 310-08 | | | 0 | 0 | 0 | | 0 | 100 | | 0 | 30 | AS | | 23.93 | 0.73 |  |
| - | Siriema AS 1 | | | 0 | 0 | 0 | | 0 | 0 | | 100 | 50 | AS | | 30.06 | 0.8 |  |
| MG 0291 | HT UFV 379-07 | | | 0 | 0 | 0 | | 0 | 0 | | 100 | 50 | AS | | 31.97 | 0.81 |  |
| MG 0364 | HT UFV 442-42 | | | 0 | 0 | 0 | | 0 | 100 | | 0 | 30 | AS | | 12.41 | 0.68 |  |
| - | IPR 100 | | | 0 | 0 | 0 | | 0 | 100 | | 0 | 30 | AS | | 24.2 | 0.67 |  |
| MG 0274 | HT UFV 377-05 | | | 0 | 0 | 0 | | 100 | 0 | | 0 | 12.1 | S | | 6.75 | 0.66 |  |
| - | Pau Brasil MG1 | | | 0 | 0 | 0 | | 0 | 50 | | 50 | 33 | AS | | 16.75 | 0.63 |  |
| - | Catuaí Vermelho IAC 144* | | | 0 | 0 | 0 | | 0 | 0 | | 100 | 50 | AS | | 32.02 | 0.62 |  |
| MG 0158 | Maragogipe | | | 0 | 0 | 100 | | 0 | 0 | | 0 | 8.3 | MS | | 4.12 | 0.61 |  |
| MG 0672 | CV x S12 Kaffa UFV 331-02 | | | 0 | 0 | 0 | | 0 | 100 | | 0 | 30 | AS | | 26.05 | 0.61 |  |
| - | IPR 103 | | | 0 | 0 | 100 | | 0 | 0 | | 0 | 7.6 | MS | | 3.2 | 0.6 |  |
| MG 0664 | CV x CIFC H276/2UFV 310-14 | | | 0 | 0 | 0 | | 0 | 0 | | 100 | 50 | AS | | 27.54 | 0.6 |  |
| MG 0303 | HT UFV 427-09 | | | 0 | 0 | 0 | | 0 | 75 | | 25 | 31.5 | AS | | 12.56 | 0.59 |  |
| - | Arara | | | 0 | 0 | 0 | | 0 | 100 | | 0 | 30 | AS | | 18.46 | 0.57 |  |
| MG 0330 | HT UFV 437-03 | | | 0 | 0 | 0 | | 0 | 50 | | 50 | 33 | AS | | 15.05 | 0.55 |  |
| - | Topázio MG 1190 | | | 0 | 0 | 0 | | 0 | 100 | | 0 | 30 | AS | | 12.58 | 0.53 |  |
| - | Catiguá MG1 | | | 0 | 0 | 0 | | 0 | 0 | | 100 | 36.1 | AS | | 15.52 | 0.52 |  |
| - | IPR 102 | | | 0 | 0 | 0 | | 0 | 0 | | 100 | 50 | AS | | 26.58 | 0.52 |  |
| MG 0331 | HT UFV 437-06 | | | 0 | 0 | 0 | | 0 | 100 | | 0 | 30 | AS | | 19.88 | 0.51 |  |
| - | Acauãnovo | | | 0 | 0 | 0 | | 100 | 0 | | 0 | 15.1 | S | | 6.96 | 0.5 |  |
| MG 0486 | K 7 x HT UFV 358-05 | | | 0 | 0 | 0 | | 0 | 100 | | 0 | 30 | AS | | 25.59 | 0.45 |  |
| MG 0506 | F 840 x HT UFV 457-34 | | | 0 | 0 | 0 | | 0 | 100 | | 0 | 30 | AS | | 19.3 | 0.44 |  |
| - | Catiguá MG 2 | | | 0 | 0 | 100 | | 0 | 0 | | 0 | 9.1 | MS | | 3.72 | 0.43 |  |
| - | MGS Aranãs | | | 0 | 0 | 0 | | 0 | 100 | | 0 | 30 | AS | | 17.34 | 0.43 |  |
| MG 0639 | CV x KP 423 UFV 308-11 | | | 0 | 0 | 0 | | 0 | 100 | | 0 | 30 | AS | | 21.16 | 0.42 |  |
| MG 0308 | HT UFV 427-55 | | | 0 | 0 | 0 | | 0 | 100 | | 0 | 30 | AS | | 21.44 | 0.42 |  |
| MG 0534 | BE 5 W W x UFV 366-08 | | | 0 | 0 | 0 | | 100 | 0 | | 0 | 14.3 | S | | 6.59 | 0.41 |  |
| MG 0416 | HT UFV 451-42 | | | 0 | 0 | 0 | | 0 | 75 | | 25 | 31.5 | AS | | 10.1 | 0.41 |  |
| MG 0296 | HT UFV 408-11 | | | 0 | 0 | 0 | | 0 | 50 | | 50 | 33 | AS | | 12.09 | 0.41 |  |
| - | Guará | | | 0 | 0 | 0 | | 0 | 50 | | 50 | 33 | AS | | 12.63 | 0.41 |  |
| MG 0342 | HT UFV 439-11 | | | 0 | 0 | 0 | | 0 | 100 | | 0 | 30 | AS | | 11.35 | 0.4 |  |
| MG 0581 | Dilla Alghe x HT UFV 400-06 | | | 0 | 0 | 0 | | 0 | 50 | | 50 | 33 | AS | | 11.06 | 0.39 |  |
| MG 0873 | CIFC HW 26/1-7 x CIFC H 371/5 UFV 333-12 | | | 0 | 0 | 0 | | 0 | 100 | | 0 | 30 | AS | | 23.19 | 0.39 |  |
| - | Oeiras MG 6851 | | | 0 | 100 | 0 | | 0 | 0 | | 0 | 4.6 | PR | | 2.55 | 0.38 |  |
| MG 0276 | HT UFV 376-57 | | | 0 | 0 | 100 | | 0 | 0 | | 0 | 8.3 | MS | | 3.87 | 0.37 |  |
| MG 0415 | HT UFV 451-41 | | | 0 | 0 | 0 | | 0 | 75 | | 25 | 31.5 | AS | | 12.37 | 0.37 |  |
| MG 0367 | HT UFV 442-108 | | | 0 | 0 | 0 | | 100 | 0 | | 0 | 15.1 | S | | 7.58 | 0.36 |  |
| MG 0311 | HT UFV 428-02 | | | 0 | 0 | 0 | | 0 | 25 | | 75 | 34.5 | AS | | 16.23 | 0.35 |  |
| MG 0669 | CV x CIFC H 276/2 UFV 310-53 | | | 0 | 0 | 0 | | 0 | 100 | | 0 | 30 | AS | | 25.16 | 0.32 |  |
| MG 0306 | HT UFV 427-24 | | | 0 | 0 | 0 | | 0 | 50 | | 50 | 33 | AS | | 11.53 | 0.29 |  |
| h^2^_m_ 0.98* | | | h^2^_i_ 0.54±0.04* | c^2^_perm_ 0.00 | | | | *M_O_* 14,51 | | | *M_S_* 4.33 | | | | SG (%) -70.16 | | |
